# Approaching Small Neuroendocrine Tumors with Radiofrequency Ablation

**DOI:** 10.3390/diagnostics13091561

**Published:** 2023-04-27

**Authors:** Gemma Rossi, Maria Chiara Petrone, Andrew J. Healey, Paolo Giorgio Arcidiacono

**Affiliations:** 1 Pancreato-Biliary Endoscopy and Endosonography Division, Pancreas Translational and Clinical Research Center, San Raffaele Scientific Institute IRCCS, Vita Salute San Raffaele University, 20132 Milan, Italy; 2 Department of Clinical Surgery, University of Edinburgh, Royal Infirmary of Edinburgh, Edinburgh EH16 4SA, UK

**Keywords:** endoscopic ultrasound, radiofrequency ablation, neuroendocrine tumors

## Abstract

In recent years, small pancreatic neuroendocrine tumors (pNETs) have shown a dramatic increase in terms of incidence and prevalence, and endoscopic ultrasound (EUS) radiofrequency ablation (RFA) is one potential method to treat the disease in selected patients. As well as the heterogeneity of pNET histology, the studies reported in the literature on EUS-RFA procedures for pNETs are heterogeneous in terms of ablation settings (particularly ablation powers), radiological controls, and radiological indications. The aim of this review is to report the current reported experience in EUS-RFA of small pNETs to help formulate the procedure indications and ablation settings. Another aim is to evaluate the timing and the modality of the radiological surveillance after the ablation. Moreover, new studies on large-scale series are needed in terms of the safety and long-term oncological efficacy of RFA on these small lesions.

## 1. Introduction

In the last few years, pancreatic neuroendocrine tumors (pNETs) have shown a notable increase in terms of incidence and prevalence; this is due to the increasingly widespread use of high-resolution imaging modalities [1,2,3]. The classification of pNETS is based on their capacity to secrete hormones and cause a syndrome (functional, F-pNETs) or not (non-functional, NF-pNETs) and on their Ki-67 expression [4,5,6].

Despite the fundamental role of pancreatic surgery as a definitive therapy, its complications remain frequent, with post-operative pancreatic fistulas in 14–58% of cases, delayed gastric emptying in 15–18%, post-operative bleeding in 1–7%, and an in-hospital mortality rate of 4–6% (depending on the surgical resection type) [7]. In this context, a minimally invasive locoregional ablative technique such as endoscopic ultrasound (EUS)-guided radiofrequency ablation (RFA) could have a prominent role in disease control in selected patients.

Many factors can influence the decision on the management of pNETs: patient-related risk factors (comorbidities, post-operative complications, life expectancy, and post-operative endocrine and exocrine insufficiency) and tumor-related risk factors (functional or not functional disease, tumor stage, lesion number, size, grading, and lesion location), which are also closely related to the neoplasia progression risk.

### 1.1. Epidemiology and Clinical Features of pNETs

pNETs are classified as pancreatic tumors, but in contrast to pancreatic adenocarcinoma (which is classified as exocrine neoplasia), they are defined as endocrine neoplasia. The endosonographic features are also different between these two types of pancreatic tumors; thus, pancreatic adenocarcinoma appears as hypoechoic and hypovascular solid lesions with an infiltrative mass, whereas pNETs typically appear as homogeneous, hypoechoic, and hypervascular (solid or sometimes partially cystic) lesions with defined margins with respect to the surrounded structures.

pNETs are classified mainly by their ability to secrete hormones (F-pNETs) which cause a specific syndrome (gastrinoma and insulinoma, for example, are the most frequent). The remainder are classified as NF-pNETs in the absence of hormone secretion by the tumor.

The frequency of both is increasing, and 60–90% of diagnosed pNETs are NF-pNETs due to their relatively indolent nature and slow growth, which cause a delay in the onset of symptoms.

Conversely, F-pNETs are diagnosed in earlier phases of the disease due to the hormone secretion causing a clinical syndrome. The most common syndromes are due to insulin (insulinoma) and gastrin secretion; the latter causes Zollinger–Ellison syndrome (featuring pain, diarrhea, ulcers, and esophageal symptoms). Established rare syndromes are caused by vasoactive intestinal peptide (VIP) secretion or somatostatin (somatostatinoma), ACTH (ACTHoma), serotonin (carcinoid syndrome), or PTHrpP (PTHrpP-oma, causing hypercalcemia) secretion. Finally, extremely rare syndromes are due to renin, luteinizing hormone, erythropoietin, insulin-like growth factor II, CCK, and GLP-1 secretion.

### 1.2. Pathological Features and Prognosis of pNETs

In general, several factors can influence the prognosis of pNET disease, including:

- The presence of calcifications at a preoperative computed tomography (CT) scan [8], which correlate to the grade or the degree of tumor differentiation and the presence of lymph node metastases in the case of well-differentiated pNETs.
- The presence of metastases (hepatic or extra-abdominal) is an important predictor of survival, regardless of the tumor grading (and the Ki-67 index; see below) [9].
- The Ki-67 index (as percentage %), which describes the tumor histology, is the best prognostic parameter to establish the likelihood of tumor progression [10]; in addition, the size of the lesion is correlated to both the potential progression risk and the Ki-67%.
- The presence and the number of lymph nodes involved in the disease, as well as the ratio between the positive lymph nodes and the total examined lymph nodes, are important predictors of recurrence after surgery [11,12]. This supports the role of systematic peri-tumoral lymphadenectomy during surgery [13].
- The absence of symptoms is associated with a significantly better outcome regardless of the tumor stage [10,14].
- The presence of a genetic syndrome (e.g., MEN1) is related to the presence of multiple lesions and the long-term effects of the disease, regardless of the specific pancreatic pathology (clinical syndrome of gastrinoma or insulinoma, hyperparathyroidism and renal failure, thymic tumors, and duodenopancreatic tumors) [4].
- The size of the tumor, which correlates to potentially malignant proliferation.

Numerous recent studies have established the importance of the different classifications and the grading systems for pNETs (WHO 2010, ENETs, AJCC/UICC) [15,16,17], and both the classification and the grading systems have a demonstrated a prognostic and therapeutic role [16,18,19,20,21].

The first WHO classification in 2010 contains morphologic and dimensional criteria about tumor differentiation and adds the grading system information, which is based on the tumor proliferative activity [22]. The grading system was proposed by ENETS [23,24] and recommended by the WHO in 2010 and is represented by the grade of differentiation of tumor cells on the basis of the mitotic count (at a high power field HPF) and the Ki67% index, using the MIB1 monoclonal antibody staining applied to histological or cytological specimens. The grading is distinguished as G1, G2, and G3, as reported in Table 1.

Following this distinction in the WHO 2010 classification, pNETs are consequently classified as NETs: G1, G2, G3, mixed adenoneuroendocrine carcinoma (MANEC), or hyperplastic and preneoplastic lesions.

It has to be highlighted that in pancreatic NETs, there is a univariate relationship between tumor size and survival [25,26,27,28], but tumor dimension is a fundamental parameter considered in the choice to undergo pancreatic resection [29,30,31,32]. 

For this reason, other staging systems have been proposed, such as the ENETS and the UICC/AJCC site-specific TNM staging [22,23,26,33,34]. These two classification systems differ in the definition of T-parameters and the pertinent tumor stages: the differences are reported in Table 2.

Together with tumor grading (G1, G2, or G3 on the basis of the Ki67% range) and the extension of the lesions, the tumor size has a key role in pNET management, especially in case of NF-pNETs. The size criteria in NF-pNETs help to inform the most appropriate follow-up, the pancreatic resection or a systemic treatment. Several studies have demonstrated a direct correlation between small size (<2 cm) and lower malignancy potential, and this kind of lesion usually goes to a clinical and radiological follow-up if the histopathological grading obtained through endoscopic ultrasound (EUS) and fine-needle aspiration (FNA) is low (G1) [35,36,37,38].

### 1.3. Endoscopic Ultrasound Role

It has been demonstrated that EUS is the more sensitive method with which to diagnosis a pancreatic lesion, with the possibility to perform an FNA or a fine-needle biopsy (FNB) to increase the diagnostic accuracy of pNETs and to establish the consequent management.

As already cited, the exact size of the pNET is imperative to establish as it will often determine the next stage of surgical/therapeutic management and the role and interval of surveillance. EUS is the most sensitive exam for the diagnosis of pancreatic cystic or solid lesions, and it also allows a highly sensitive description of echoic specific features (vascularization, contrast-enhancement, and elastography). Hence, it is integral to the management of the patient. EUS also allows accurate anatomical description when surgical resection is planned, including, importantly, the distance of the lesion from the main pancreatic duct or the presence of pathological lymph nodes.

**Figure 1:**
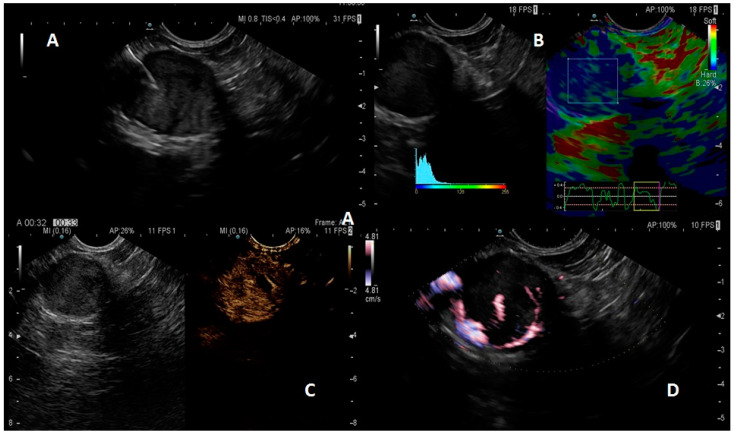
Pancreatic neuroendocrine tumor (pNET) aspect. B-mode (**A**): hypoechoic lesion with well-defined margins; qualitative and semi-quantitative elastography; (**B**): pNET as a stiff, blue lesion; pNET at intravenous contrast; (**C**): early hyperenhancement of the contrast; pNET aspect at power Doppler (**D**), with a peripheral vessel capsule.

In general, a well-differentiated pNET appears in EUS as a hypoechoic lesion with well-rounded margins, hypervascular in Doppler imaging, with a vascular rim at the margins of the lesion, and rigid in elastography (an applied software which can assess tissue stiffness). After intravenously contrast-enhanced EUS (CE-EUS), a pNET shows a fast uptake of the contrast and a rapid consequent wash-out (Figure 1).

Finally, as already described above, the cytological or histological material obtained by EUS-FNA or FNB has a key role in establishing the prognostic evaluation of the lesion (grading, Ki67%).

An improvement in the EUS needles used, in terms of needle tip design, materials, and flexibility, has been observed over the last decade, with the final aim of obtaining more useful material to enable immunohistochemistry evaluation.

The purpose of the most commonly used EUS needles is to obtain a core biopsy in order to use the material for subsequent immunohistochemistry reactions. FNB needle designs are varied in terms of size (19, 22, and 25 Gauge (G)), materials, and tip designs. The tips of “Franseen needles” are designed with three symmetrical cutting surfaces with fully formed heels; “Menghini needles” are designed for receiving tissue into the needle with a Menghini bevel; “shark needles” have a tip designed like the teeth of a shark, which are very sharp; and, finally, “Lancet needles” are designed with a blunt tip and are similar to FNA needles, but they are usually large in caliber to obtain a core (19 G).

FNA needles can be used, especially if a cytopathologist is present in the room (rapid on Site evaluation (ROSE) of the sample) who can confirm directly at a microscope the presence of representative material for a pNET diagnosis.

### 1.4. EUS-Guided Radiofrequency Ablation

EUS has increasingly been used in a therapeutic role during the last decade. The precision with which a pNET can be isolated can facilitate minimally invasive (EUS-guided) treatment methods, and the close proximity of the EUS probe to the pancreas, coupled with the possibility to insert therapeutic devices into the operative channel of the echoendoscope, has contributed to the development of EUS-guided therapies for pancreatic focal lesions.

EUS radiofrequency ablation (RFA) represents a therapeutic modality for the treatment of selected pNETs, and there have been multiple case series reported with promising results. RFA uses thermal ablation to induce coagulative necrosis within the tumor nodule by generating focally high temperatures. The first RFA experiences were reported in percutaneous or intraoperative (i.e., surgical) case series.

The most used EUS-RFA system (STARmed Co., Ltd., Koyang, Korea) consists of a specific probe designed like an EUS needle (inserted into the endoscopic channel) with a monopolar electrode on the distal tip delivering the thermal energy and a radiofrequency generator above, through which the ablation power can be controlled. A peristaltic pump connected to the needle allows a continuous, chilled saline solution perfusion to prevent tissue charring around the probe. Power is delivered from the generator and is often preplanned such that the system can automatically modulate (increase or decrease) it if tissue impedance quickly increases during ablation to maximize the ablation area. The electrode is available in different sizes/lengths on the basis of the size of the pNETs being treated.

The second available RFA system consists of a monopolar radiofrequency probe [Habib™ EUS-RFA catheter, Emcision Ltd., London (CE Marked), London, UK] with a caliber of 1 Fr (0.33 mm, 0.013″) and a working length of 190 cm, which can be placed through a 19 or 22 G FNA needle. The wire can be connected to a RITA generator (Electrosurgical RF Generator) which can release the energy and can automatically adjust the wattage to maintain the optimal temperatures during the ablation. As the Habib probe is a monopolar device, it requires the use of a patient grounding/diathermy pad.

RFA in pNET treatment can be useful in symptomatic patients who are not eligible for surgery (due to poor performance status or patient choice) or in selected cases of NF-pNETs evaluated by a multidisciplinary team (e.g., borderline lesions or syndromic patients with an increase in multifocal tumors).

EUS-RFA is reported widely in the published literature, albeit without a standardization of such parameters as the power setting used for ablation or pre-ablative and post-ablative imaging controls, which are fundamental to the evaluation of the final necrosis effect of the ablation.

The aim of this review is to report and summarize the heterogenous data from the previously published studies on the EUS-RFA of small-pNETs to try and find a common procedure indication and ablation setting. Another aim is to evaluate the timing and method of the radiological controls after the reported ablation and to find a rationale for the timing of pre- and post-ablative imaging.

## 2. Previous EUS-RFA Experiences in Small pNETs

The EUS-RFA systems were set following the results of pre-clinical ex vivo and in vivo models.

The first animal in vivo study [39] with the STARMED system was published in 2012. A pancreatic RFA was performed in 10 adult pigs at 50 W of power, and the treatment was applied for 5 min. The power and the time setting were decided following previous ex vivo tests on animal liver, which established that the most effective power was 50 W [40]. A well-demarcated ablated zone, consisting of a central coagulative necrosis, was obtained and distinguished from the surrounded healthy pancreatic parenchyma by a fibrotic rim. Non-pathological ablated areas were reported (23 ± 6.9 mm) as a result of RFA but with only macroscopic measurements. In 3/10 pigs, adverse events were observed (retroperitoneal fibrosis and adhesions).

In 2015, Armellini et al. [41] described the first experiences of EUS-RFA applied to a G2 pNET (size 20 mm) in the pancreatic tail of a 76-year-old man who had not consented to surgery. The RFA was applied with an 18 G RFA needle with a 10 mm active tip in a single session (two RFA applications), and no complications were evidenced (administered rectal indomethacin to prevent acute pancreatitis). A CT scan control and EUS at 1 month evidenced a necrotic area inside the ablation zone.

In 2016, Lakhtakia et al. [42] treated three insulinomas of patients not eligible for surgery with the same system. A 19 G RFA needle was used at 50 W of power for a time period that was dependent on the tissue impedance (the generator can monitor the power and impedance during the ablation and stop the energy delivery if tissue impedance goes beyond a certain value). All the patients of the series showed symptom relief, which was maintained at 11–12 months. No specific radiological protocols were employed to appraise the immediate post-procedural and long-term effects of RFA.

In 2018, Barthet et al. [43] reported a larger series of pNETs in which 14 lesions in 12 patients (<2 cm and 17 pancreatic cystic lesions (PCLs)) not eligible for surgery were treated with EUS-RFA using an 18 G cooling needle with the application of 50 W of power. Three adverse events occurred in total, including those of the first patients, who developed pancreatitis, bowel perforation, and/or main pancreatic duct stenosis. Thereafter, this was managed by power reduction, protocol change with the administration of antibiotics, and rectal diclofenac. Technical success was achieved in all NETs. At a 6-month follow-up, nine NETs disappeared or showed complete necrosis, and one decreased in diameter by >50% (71% of response), and in four NETs, the EUS-RFA was considered to have failed. At a 1-year follow-up, 12 NETs had completely disappeared, and the treatment of two pNETs failed.

The same group reported the long-term efficacy of EUS-RFA treatment [44] after two years; the treatment showed an efficacy of 87.5% with two failures: one patient showed a lesion increase after refusing the second session of EUS-RFA, and one patient received the second RFA session after a lesion increase, with appearance at 3 years of a secondary liver lesion.

In 2018, Choi et al. [45] showed the results of EUS-RFA on 10 patients at 50 W of power. Seven of the patients had an NET diagnosis. During 13 months of median follow-up, they reported a radiologically complete response in 7/10 patients. The NETs had a median size of 20 mm, and two adverse events occurred: one moderate and one mild. They concluded that more sessions were necessary with this procedure.

In 2019, Oleinikov et al. [46] presented their series of 18 patients with pNETs, 7 patients with insulinomas and 11 with NF-NETs (total lesions 27); they were treated with a 19 G EUS-RFA needle at 50 W. In 12/18 patients, a CT scan was performed 1 day after the RFA in order to establish early complications. In all the patients in this series, imaging was performed at 3 and 6 months after treatment with a CT scan, EUS, and a positron gallium 68-1,4,7,10- tetraazacyclododecane-1,4,7,10-tetraacetic acid-octreotate (68Ga-DOTATATE) positron emission tomography (PET) to assess the metabolic response. The mean lesions had a size of 14.3 ± 7.3 mm, and six older patients (>80 years) had multiple lesions. In the patients with F-pNETs, a clinical response of hypoglycemic symptoms was assessed as a EUS-RFA response within 1 h post-procedure; the normalization of glucose levels occurred within 24 h, and the clinical response was maintained at 9.7 ± 5.6 months. Technical success defined as typical “postablative changes” on the surveillance imaging and was achieved in 26 out of 27 lesions. A radiologically complete response was achieved in 17/18 patients and in 26 out of 27 treated lesions. Changes in the vascularity of the treated lesions and the central necrosis were demonstrated by CT scan in 12/12 patients one day post-procedure. There were two patients with mild pancreatitis post-treatment. No major complications occurred, and no clinically significant recurrences were observed during a mean follow-up period of 8.7 ± 4.6 months.

Then, in 2020, de Nucci et al. reported on [47] a series of 10 patients with pNETs (11 lesions and five hypoglycemia symptoms) of ≤20 mm (mean size 14.5 mm) who were treated with RFA and received a follow-up at 1 year; the patients showed a complete ablation of the lesions in a single EUS-RFA session, with a mean of 2.3 treatment applications per session. A 19 G needle (with active distal part of 5 or 10 mm) was used at, in this specific case, a power of 20 W, which was lower than the previous procedure. The author stated that by using 20 Watts of power for 10 to 25 s (working with impedance) and a 5 or 10 mm exposed active tip (according to the size of the tumor) an ablation area of about 10 to 25 mm after each application could be produced. The patients received a follow-up with CT scans at 6 and 12 months, with a complete lesion resolution. No major adverse events occurred but two mild cases of abdominal pains were resolved with analgesics. The patients received prophylactic antibiotics and indomethacin to prevent pancreatitis (2 mm of length was maintained respect to the duct–lesion distance).

In 2022, Marx [48] treated the symptoms of seven insulinomas with success, with immediate hypoglycemia relief after only one single treatment session; in six out of seven patients, a complete radiological response was evidenced and remained asymptomatic in a median follow-up of 21 months. Three patients showed minor adverse events and one elderly patient showed a 15 cm retrogastric collection 15 days after treatment and died 1 month after EUS-RFA. After the cited results, the authors concluded that data on the long-term survival and effectiveness were needed.

In 2022, Younis et al. [49] treated seven pNETs together with five PCLs with a 19 G needle (with a needle active part of 10 mm) at 50 W of power. The patients received prophylactic antibiotics and diclofenac for acute pancreatitis prevention. A 6-month EUS was planned for the follow-up, along with an assessment of the efficacy with imaging and metabolic control at 12 months. The median size of the pNETs was 8.9 mm and the median number of applications was 3.33. Three percent of the patients were non-responders. The single insulinoma observed maintained the response at 1 year. Three post-procedural adverse events occurred; 8.3% had mild acute pancreatitis, and 16.7% had abdominal pain.

In 2022, Rossi et al. [50] reported the EUS-RFA of three elderly symptomatic non-surgical patients with insulinoma. The lesions were treated at 30 W with a 19 G needle with a 5 mm or 10 mm active part; there were two RFA sessions due to a failure in the first procedure. The settings were decided on the basis of previous ex vivo animal and human tests. All the patients were studied at 48–72 h with a CT scan in order to assess the presence of complications and to report the size of induced coagulative necrosis inside the lesions. Three out of three patients responded with symptom resolution after up to 24–27 months of follow-up. Two patients refused long-term follow-up (one patient was not a candidate for contrast-enhanced CT due to poor renal function) and an MRI was performed on the third patient at 14 months, with lesion disappearance.

The second RFA system reported (a Habib™ probe inserted into a 22 G needle and connected to a generator) was used by Pai et al. in 2015 [51] in a multi-center pilot study. The probe was used in eight patients (six PCLs and two pNETs) at 5–25 W for a mean application time of 90 s. The EUS-RFA technique success was 100% (complete resolution of two lesions). As an intravenous contrast, the pNETs showed a central not-vascularized necrosis after the treatment. No complications were described (just two self-limiting abdominal pains).

## 3. Discussion

### 3.1. EUS-RFA Indications

As reported in the cited case series, EUS-RFA was applied to small pancreatic lesions in patients who refused or were not eligible for major pancreatic surgery, which is the first therapeutic choice in small single lesions and fit patients. The size of pNETs commonly correlate to the grading of the lesions, and these patients mainly had small G1 single lesions with limited true indications for surgical intervention. Current consensus guidelines (4–6) advise a non-operative management strategy for asymptomatic sporadic NF-pNET ≤ 2 cm. A conservative approach seems to be safe as the majority of the observed tumors did not show any significant changes during follow-up.

In cases of F-pNET (commonly insulinoma), the approach has to be different due to the symptoms and clinical syndrome induced. In such cases, a minimally invasive endoscopic procedure such as the EUS-guided RFA can offer elderly/frail patients (not eligible for surgery) a possibility of symptom resolution. Unfortunately, the current series reported in the literature are small due to the relative rarity of the disease, but the results are encouraging. The long-term efficacy of symptom control is not reported and longer-term studies and follow-up data are required.

All patients with both F- and NF-pNETs being considered for treatment with EUS-RFA should be evaluated in a multidisciplinary meeting at which the many factors (procedural and patient-related) should be considered. EUS-guided RFA represents a minimally invasive treatment, which can permit a shorter hospitalization, avoiding all the complications of a pancreatic surgery, though surgery remains the mainstay treatment for certain pNETs and can provide significant benefits to carefully selected patients in terms of long-term and disease-free survival [52]. Despite curative efficacy, pancreatic surgery is associated with significant short- and long-term adverse events such as pancreatic fistulas, delayed gastric emptying, hemorrhage, and in-hospital mortality. Hence, MDT discussions should entail balancing the risk of under- or overtreatment on the basis of the patients’ comorbidity, risk of post-operative death, life expectancy, tumor location, risk of post-operative fistula and post-operative morbidity, and risk of long-term exocrine and/or endocrine insufficiency [53].

On the other hand, the possibility of a minimally invasive and less-expensive method to control this disease could be an alternative in a defined demographic setting.

### 3.2. RFA Setting

Another important point is the setting applied during EUS-RFA, and all the series showed significant heterogeneity with respect to this issue. Some authors performed treatment using a high ablation power of 50 W and a charring effect was created around the needle, while other authors, in contrast, used 30 W or 20 W to employ heat diffusion for major therapeutic effect.

One such group used a preset 30 W of power to treat pNETs, on the basis of ex vivo animal and human tests [50]. The first tests applied different powers (40, 30, 20, and 10 W with a 19 G probe with 1 cm of distal electrode) on ex vivo samples of pig liver in order to standardize the method and the RFA settings. The size of the macroscopic ablated area negatively correlated with the ablation power (R −0.74), with the largest area obtained at 10 W [54]. The coagulative necrosis obtained with RFA (confirmed by a specialist pancreatic pathologist) did not differ among the ablation settings (mean size 3.25 mm). After this experience, other tests were performed on human surgical samples of pancreatic adenocarcinoma (PDAC), treating three groups of samples ex vivo (probe 19 G with 1 cm active part) at 10, 30, and 50 W [55]. Eighty percent of the specimens showed coagulative necrosis at histology consisting of a few millimeters (the majority had a size of 5.7 ± 3.9 mm at 10 W; those not statistically significant with respect to necrosis were obtained at 30 and 50 W). This setting may not be practical to apply in the context of NETs that are often represented by tissues which are more “soft”, but the work represents an attempt at method standardization.

### 3.3. Pre-Operative and Post-Operative Purpose of Management

Moreover, the timing of the radiological controls after the EUS-RFA treatments was heterogeneous among the studies. EUS-RFA can induce a coagulative necrosis inside the lesions and, being a thermal treatment, it can also cause complications such as hemorrhage, intestinal adhesions, acute pancreatitis, and main pancreatic duct stenosis.

Therefore, a relatively close imaging control should be planned at 24–72 h with respect to the RFA in order to evidence the size of coagulative necrosis obtained and exclude and/or manage post-procedural complications (such as bleeding, acute pancreatitis, and early collections). Moreover, a long-term follow-up should be planned in order to control the disappearance of the lesion or the residue of it and to rule out late complications, such as pancreatic duct stenosis, intestinal adhesions, and late collections. A CT or MRI scan at 6 months and then at 12 months may represent an adequate follow-up, but more prospective studies are needed to assess this. In the case of F-pNETs, it is easier, due to the clinical syndrome which can guide the response to RFA.

Further studies may also assess whether radiological studies are able to accurately compare the necrotic area obtained with RFA to the surrounding healthy tissue. No such comparison is evident in the aforementioned studies, though the Italian group (previously cited [55]) tried to analyze the coagulative necrosis obtained inside PDAC samples through contrast-enhanced CT scan parameters. Qualitative and quantitative parameters were extracted to establish the RFA response and lesion fibrosis content.

An open question also concerns the utility of metabolic imaging to establish the effect of RFA, and one study used the 68Ga-DOTATATE-PET. Moreover, contrast-enhanced EUS (CEH-EUS) undoubtedly has a utility in describing both pre- and post-ablation vascularization in the lesion, as described by Choi et al. [56] with regard to 19 patients (13 NF-pNETS) with solid abdominal tumors who underwent to CEH-EUS and endoscopic RFA. The extent of the ablation was assessed by CEH-EUS at 5 to 7 days after RFA, and additional RFAs were performed under CEH-EUS guidance. Seven patients showed the disappearance of intratumoral enhancement on CEH-EUS after the first session and twelve showed a residual contrast enhancement (a sign of an incomplete ablation (further treated with additional CEH-EUS-guided RFA)). A radiologically complete response was observed in 68.4%. The authors concluded that the application of CEH-EUS for RFA could be helpful in assessing the early treatment response and, eventually, in the targeting of the residual tumor.

### 3.4. EUS-RFA Limitations

The technique needs a relatively long learning curve in terms of diagnostic and operative EUS ability, and the method should be applied in lesions evaluated by a high-volume center by a multidisciplinary team expert in pancreatic diseases and NETs. These conditions are not present in a low-volume peripheral hospital; therefore, the method is not so easily reproducible. Moreover, the management of possible complications should be managed by a specialist referral center with expertise in endoscopic (pancreatic duct dilation or hemostatic techniques) and EUS-guided therapeutic procedures (drainages).

In summary, the technique needs to be improved in terms of the ablation setting, which needs to be standardized: the absence of standardized results does not facilitate the method’s diffusion.

## 4. Conclusions and Future Directions

On the basis of the results reported in the literature, EUS-RFA could be an effective and relatively safe procedure for pNET treatment, and its indication should be discussed by a multidisciplinary team of pancreatologists, especially in the case of functional tumors.

The standardization of the method in terms of device power settings and the radiological protocols for the monitoring of RFA results is necessary, and more large-scale results are needed to determine the long-term efficacy.

## Figures and Tables

**Table 1:** Definition of tumor grading for neuroendocrine neoplasms of the digestive system according to the WHO 2010 classification on the basis of mitotic count as high field (HPF) and Ki67% index.

Grade	Mitotic Count	Ki67% Index
G1	mitotic count is 2 per 10 high field (HPF)	≤3%
G2	mitotic count is 2–20 per 10 HPF	4–20%
G3	mitotic count is >20 per 10 HPF	>20%

**Table 2:** Differences in pancreatic neuroendocrine tumor pT definition according to AJCC/UICC/WHO and ENETS TNM staging systems.

T	AJCC/UICC/WHO	ENETS
T1	lesion confined to the pancreas with a size < 2 cm	tumor confined to pancreas and <2 cm in size
T2	lesion confined to the pancreas with a size > 2 cm	tumor confined to the pancreas with a size between 2 and 4 cm
T3	lesion with a peripancreatic spread without the involvement of superior mesenteric artery	lesion confined to pancreas with a size > 4 cm or with the invasion of duodenum/bile duct
T4	lesion involves the coeliac axis or the superior mesenteric artery	invasion of adjacent organs (stomach, spleen, colon, adrenal gland) or the wall of large vessels (coeliac axis or the superior mesenteric artery)

## Data Availability

Not applicable.
